# Percutaneous Computed Tomography (CT)-Guided Localization with Indocyanine Green for the Thoracoscopic Resection of Small Pulmonary Nodules

**DOI:** 10.3390/jcm12196149

**Published:** 2023-09-23

**Authors:** Emanuele Voulaz, Veronica Maria Giudici, Ezio Lanza, Edoardo Bottoni, Umberto Cariboni, Alessandro Crepaldi, Giuseppe Ferrillo, Giuseppe Marulli, Marco Alloisio, Giuseppe Mangiameli, Alberto Testori

**Affiliations:** 1 Division of Thoracic Surgery, IRCCS Humanitas Research Hospital, Via Manzoni 56 Rozzano, 20089 Milan, Italy; 2 Department of Biomedical Sciences, Humanitas University, Via Rita Levi Montalcini 4 Pieve Emanuele, 20090 Milan, Italy; 3 Department of Diagnostic and Interventional Radiology, IRCCS Humanitas Research Hospital, Via Manzoni 56 Rozzano, 20089 Milan, Italy

**Keywords:** pulmonary nodule, video-assisted thoracoscopy surgery, computed tomography, indocyanine green

## Abstract

Background: The identification of small lung nodules is challenging during mini-invasive thoracic surgery. Unable to palpate them directly, surgeons have developed several methods to preoperatively localize pulmonary nodules, including the computed tomography-guided positioning of coils or metallic landmarks (hook wire) or bronchoscopic marking. Methods: We present a series of patients scheduled for the video-assisted thoracoscopic sublobar resection of small pulmonary nodules, in which we performed preoperative percutaneous computed tomography (CT)-guided nodule localization through the injection of a mixture of indocyanine green and human albumin. Results: A total of 40 patients underwent a preoperative CT-guided injection of indocyanine green followed by VATS resection within 24 h. Patients tolerated the procedure well, no pain medication was administrated, and no complications were observed during the marking procedure. All pulmonary nodules were easily detected and successfully resected. Conclusion: the near-infrared dye marking solution of indocyanine green (ICG) with diluted human albumin was safe, effective, and easy to perform. The ICG solution has the potential to facilitate the accurate localization and resection of pulmonary nodules during VATS surgery, avoiding the risk of marker displacement/migration.

## 1. Introduction

Since the beginning of the 2000s, with the introduction of lung cancer screening with computed tomography (CT), small sub-centimeter lung lesions have frequently detected, especially with ground-glass opacities, which are known to have a high malignant potential [1,2]. Otherwise, patients affected by extra-thoracic malignancy may develop infra-centimetric lung nodules during surveillance that may require a multidisciplinary treatment, including surgery aimed at improved survival (e.g., colorectal cancer) [3,4].

Mini-invasive surgery is considered the approach of choice for the resection of small lung nodules located less than 3 cm from the visceral pleura [5] because it results in minimal postoperative morbidity and mortality, along with less pain and a better quality of life than with open thoracotomy [6].

Although the localization of these lesions may sometimes be challenging during video-assisted thoracoscopic surgery, to address the inability to visualize and palpate them directly, especially when no changes appear on the visceral pleura, clinicians work to preoperatively localize lung nodules [7,8,9].

Prolonged operative time, due to searching for the nodule or conversion to an unplanned open thoracotomy, are related to the inability to find the nodule through digital palpation or due to inaccurate nodule localization [10]. Additionally, inadequate tumor margins increase local recurrence rates with the potential for the microscopic spread of cancer cells from the surgical margin [11]. 

For all these reasons, thoracic surgeons have developed several methods of preoperative nodule targeting, such as CT-guided hook-thread, spiral-wire, or coil deployment [12,13,14,15,16,17,18,19,20,21]; the hook-wire localization represents the most common technique [13]. 

The success rate of localization reported in the literature is about 94–100%, [16,22,23,24], even though some complications such as iatrogenic pneumothorax, bleeding, and wire dislodgment have been described. 

Near-infrared (NIR) fluorescent imaging with indocyanine green (ICG) has been introduced for the real-time visualization of the lymphatic flow and tissue perfusion during surgery with a different concentration according to the type of use [25,26].

Indocyanine green is a negatively charged amphiphilic tricarbocyanine dye with high tissue penetration and low autofluorescence and is representative of a wide range of NIR spectroscopic dyes used in clinical practice and medical research [27]. ICG is not visible to the naked eye and does not interfere with the surgical field of view without the use of NIR light-imaging systems. It has been successfully employed in the preoperative targeting of pulmonary nodules powered by Electromagnetic Navigation Bronchoscopy [28,29] and through CT-guided preoperative marking with safe, feasible, and successful outcomes [30,31,32,33,34]. Based on this experience, we applied indocyanine green (A.P.M. Srl—Italy) diluted in human albumin to preoperatively localize lung nodules, using a CT-guided small needle technique similar to the more widely adopted hook-wire positioning. 

The purpose of this study is to confirm the safety and efficacy of CT-guided ICG staining to localize non-palpable nodules during thoracoscopic surgery. 

## 2. Materials and Methods 

We present a series of 40 patients who were scheduled for VATS sublobar resection of a nonsolid, partly solid, or solid pulmonary nodule with a maximum nodule diameter <25 mm located in the peripheral part of the lung away from the hilar structures and the great vessels of the lung. 

The main exclusion criteria were as follows: patients less than 16 years old, an anatomic location of nodule making the CT-guided radiolabeling technically difficult, patients not being willing to undergo the procedure, the inability to consent to the operation, and pregnancy. Each eligible case was discussed at the multidisciplinary lung cancer meeting, which was attended by the radiologist, oncologist, pneumologist, and thoracic surgeon.

The study was conducted at Thoracic Division of IRCCS Humanitas Research Hospital, Rozzano–Italy, from 1 April 2022 to 31 March 2023. All patients signed informed consent, agreeing to receive preoperative CT-guided ICG staining with injection of ICG. Institutional Review Board approval was obtained for the present study on 31 January 2022 (code GREEN1).

The data used in this study were retrieved from patient medical records, which included the nodule size, CT-scan characteristics (ground-glass nodules, solid, nonsolid), operation method, lesion depth from pleura, location, and histology (metastasis, non-small cell lung cancer, carcinoid, and hamartoma). Retrospective and descriptive analyses were performed. 

### 2.1. Marking Procedure

Patients were positioned prone, supine, or in lateral decubitus, depending on the shortest distance from the skin to the target. A preoperative, low-dose chest CT scan was performed (80 Kvp tube voltage, automatically modulated tube current reference: 100 mAs, 1 mm Slice Thickness, 1 mm slice interval, 0.6 s gantry rotation time, 1.2 pitch, 128 mm × 0.625 mm collimation width, 360 mm field of view Philips Brilliance 64, Germany) to confirm nodule localization, size, shape, and relationship with surrounding structures (pleura, small vessels, and chest wall).

**Figure 1:**
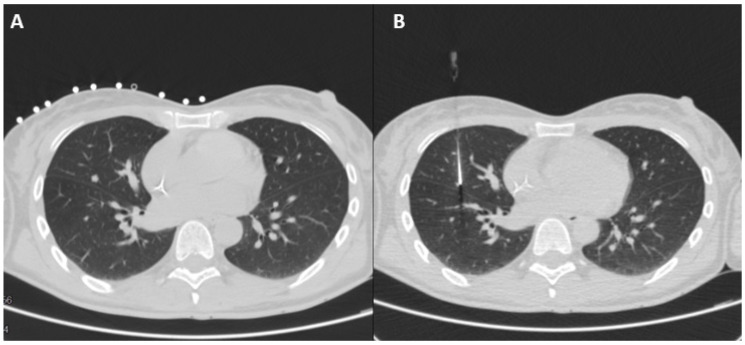
Preoperative CT-guided procedure: (**A**) localizer-computed tomography to detect the pulmonary nodule; (**B**) CT-guided percutaneous injection of indocyanine green.

Then, a real-time CT-fluoroscopy protocol (80 Kvp tube voltage, automatically modulated tube current: 100 mAs, 5 or 10 mm Slice Thickness) was started, centered on the target nodule. The operator (a skilled interventional radiologist with at least 5 years’ experience in percutaneous CT-guided biopsies) inserted a short 23 G needle along the chosen puncture track to inject 5 mL of mepivacaine chlorhydrate to achieve subcutaneous anesthesia; subsequently, a longer 10 cm spinal needle 21 G of caliber was introduced through the track down to the target nodule. The tip of the needle was placed inside or near the nodule, depending on the distance from the pleura. In the case of subpleural nodules (e.g., ≤3 mm from the visceral pleural fold), the injection was aimed at marking the underlying lung parenchyma to avoid undesirable pleural diffusion. Finally, 0.1 to 0.5 mL of a mixture of ICG powder at 50 mg/10 mL, diluted in 20 mL of human albumin 20% (2.5 mg/mL), was injected (Figure 1), and a final CT scan was performed to confirm if the positioning was satisfactory and to exclude pneumothorax, bleeding, or any other complications. We used human albumin because its adsorption of ICG results in an increased hydrodynamic diameter and has the effect of increasing fluorescence, leading to improved nodule detection. The concentration of ICG in the mixture of human albumin results from a similar study that tested this technique to identify the sentinel lymph nodes in non-small-cell lung cancer [35], and the injected volume depends on the dimension and the depth of the nodule. 

The patient was transferred bedridden outside the CT room and observed for 30 min. Oxygen saturation and systemic blood pressure were checked in real time for 30 min by a team of nurses. Then, a low-dose CT scan was repeated to assess any occurrence of pneumothorax, pulmonary hemorrhage, or air embolism. The entire procedure lasted approximately 60 min.

### 2.2. Surgery

Surgery was performed in intervals of time varying from 5 to 18 h from the marking procedure. The radiologists generally marked the lesion the same day or the afternoon prior, according to the operating list and availability of the CT room. 

The patients were conducted in an operatory room. All surgery procedures were performed under general anesthesia, and the intubation was carried out by using a double-lumen endobronchial tube to allow single-lung ventilation. All patients were placed in lateral decubitus, and the hips were located at the level of the table break and flexed. All surgical resections were conducted by using a one- or two-port VATS approach. In the case of a two-port approach, a 2 cm utility incision was performed in the IV or V intercostal space and a video port in the VII intercostal space; in those surgeries performed through a single incision, this was generally made in the IV or V intercostal space. A soft tissue retractor was positioned (Alexis wound retractor—Applied Medical, Rancho Santa Margarita, CA, USA) to allow for the introduction of the thoracoscopic instruments and the 30° camera.

**Figure 2:**
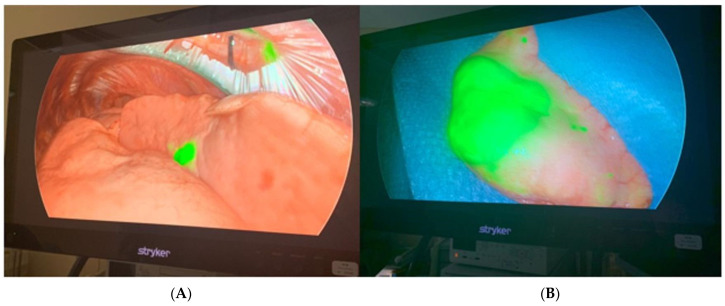
Surgical resection: (**A**) thoracoscopic near-infrared dye-marking detection with Stryker’s SPY Fluorescence technology with a 30° camera; (**B**) resected lung specimen from the surgical field.

The exploration of the pleural cavity with a 30° fluorescence camera (Stryker SPY, Kalamazoo, MI, USA) was carried out to confirm the absence of pleural effusion or other suspected lesions; this kind of camera allowed the immediate detection of the nodules previously marked with the CT-guided injection of indocyanine green (Figure 2). 

Thoracoscopic instruments were used to define the output of the lung to be resected (green area), and sublobar resections were performed by using single or multiple refills of manual staplers. The presence of a correct surgical margin was immediately verified and confirmed after specimen extraction. All resected specimens suspected to be primary lung cancer were sent for immediate histopathologic examination; in these cases, the completion of lobectomy and mediastinal lymph node dissection were performed in the same operation. 

All the resections were subsequently analyzed by the pathologist to confirm the diagnosis and to define the distance to the resection margin and the visceral pleural invasion. 

To verify the correct seal of the lung parenchyma suture, all patients, before the end of the operation, underwent a hydropneumatic test with the inclusion of the operated lung. At the end of the surgery, the chest drainage was positioned either through the video-port or through the utility incision. The camera was used to confirm the correct placement of the drainage. 

The patients were woken up in the operating room and underwent a chest X-ray before being transferred to the ward. 

## 3. Results 

### 3.1. Patient Characteristics

A total of 40 patients presenting 43 nodules were subjected to a preoperative CT-guided injection of indocyanine green (ICG) followed by VATS resection. Th simultaneous marking of nodules was performed in three patients, one had two nodules located in the ipsilateral lung (upper and lower left lobe), one patient presented synchronous bilateral lesions (right upper lobe and left lower lobe), and one patient presented two separate nodules in the same lobe. 

There were 23 males and 17 females patients, with a mean age of 62.6 years (range: 35–83 years). Twenty-nine patients had a previous cancer history, and five had a preoperative diagnosis. Twenty-two patients had no smoking history, fourteen were former smokers, and four were active smokers at the time of surgery. Respiratory function was investigated by performing spirometry; the mean value of FEV1 was 2.69 L (range 4.92–1.38). The patients’ characteristics are summarized in Table 1. 

### 3.2. Radiological Characteristics and Localization of the Pulmonary Nodules

Based on the radiological characteristics of the CT scan, pulmonary nodules were classified as solid (*n* = 36; 83.7%), sub-solid (*n* = 3; 7%), or pure ground glass (*n* = 4, 9.3%). The mean nodule diameter was 10.4 mm (range, 3–21 mm). The mean distance from the nodule to the nearest visceral pleura was 8.1 mm (range, 2–28 mm). The mean interval from the radiological localization to surgery was 365 min (range 120–990 min); in three patients, the marking procedure was performed the day before surgery. Nodules were located in the right upper lobe in nine patients (20.9%), in the middle lobe in four cases (9.4%), and six patients presented nodules in the right lower lobe (13.9%). In 6 cases, nodules were located in the left upper lobe (20.9%), and 15 patients presented the lesion in the left lower lobe (34.9%). No hemothorax, pneumothorax, or systemic air embolism was observed during the marking procedure. No complications that required invasive management occurred during the period between the radiological procedure and surgery. The procedure was well tolerated by patients, and no pain medication was administered. 

### 3.3. Operative Characteristics 

All pulmonary nodules were resected under VATS, and none of the procedures required conversion to a thoracotomy. The mean value of the surgical procedure time was 52 min (range: 22–119). Three patients (12%) who underwent an initial wedge resection underwent lobectomy and systematical lymphadenectomy according to frozen-section analysis, which confirmed a primary lung cancer diagnosis—solid adenocarcinoma. In the other seven patients with an intraoperative diagnosis of NSCLC, wedge resection and lymphadenectomy were considered as the appropriate treatment (in two patients, previously resected for previous primary NSCLC, we received an intraoperative diagnosis of squamous cell carcinoma, and in the other five patients, the final diagnosis confirmed minimally invasive adenocarcinoma). The histopathological diagnosis was formulated according to the 2021 WHO Classification of Lung Tumors [36]. 

Twenty-three patients had a final diagnosis of lung metastatic disease: in fifteen cases (34.9%), we observed metastasis from colorectal cancer—one patient had two separate nodules in two different lobes; in two cases (4.6%) of metastasis from melanoma, one patient received a diagnosis of metastasis from kidney tumor for two bilateral nodules; in one case (2.1%), metastasis was from adenoid cystic carcinoma; in one case (2.1%), metastasis was from cholangiocarcinoma; in one case (2.1%), metastasis was from an adrenal gland tumor; and in one case (2.1%), metastasis was from extragonadal germ cell tumors. Ten patients had benign lesions: there were three cases of hamartoma (7%), one case (2.1%) of localization of pulmonary sarcoidosis, and six patients (13.9%) received a diagnosis of a benign condition (three inflammatory nodule, two bone metaplasia, and one neuroendocrine cell’s hyperplasia). Detailed information on histology and surgery are listed in Table 2. 

The surgical margin of all wedge resection specimens was microscopically negative without the additional resection of lung parenchyma.

According to intraoperative findings for wedge resection, ICG was successfully injected in all patients, obtaining 100% (43 nodules) of targeting success rate; in three patients, a visceral pleural retraction was observed. After carrying out wedge resection, we used a fluoroscope to confirm the ICG fluorescence in the resected specimen. All patients who underwent this procedure showed no ICG-related adverse effects.

Patients were discharged from the hospital without complications. The median length of stay was 3 days (range 1–7 days).

## 4. Discussion

Since the spread use of low-dose CT scans for NSCLC screening programs, the detection of small lung lesions has increased [1,37]. Similarly, the surgical treatment of lung metastases is nowadays commonly considered in selected patients [38]. In the case of failed histological diagnosis through percutaneous transthoracic lung biopsy or to perform diagnostic lung resections, VATS is the most common surgical procedure of choice.

However, some pulmonary nodules cannot be seen by the naked eye and cannot be palpated by fingers during the operation, bringing further challenges to clinical diagnosis and treatment [39]. 

For these reasons, several preoperative marking procedures have been developed, aiming at the easier localization of small-sized nodules during VATS [7,10], resulting in reduced operation time.

A lot of localization techniques have been proposed and adopted to assist VATS in reducing operative difficulty and time: hook-thread, spiral wire needle, micro coil, fiducial marker, or dyes are the most common markers reported in the literature, and each tool is usually inserted inside the lung near the nodule using a CT-guided percutaneous approach with specific advantages and potential shortcomings. 

The first adopted technique, borrowed from oncological breast surgery, was the localization through a hook wire, with commonly reported drawbacks including pneumothorax, bleeding, wire dislodgement, and rare complications such as air embolism [10,13,14,15,16,17,18,19,20,21,22,23,24,25,26,27,28,29,30,31,32,33,34,35,36,37,38,39,40]. 

Asamura et al. in 1994 and Lizza et al. later in 2001 reported a procedure that provided before the injection of a metallic coil and successively the pulmonary resection under roentgenographic fluoroscopy [18,41]. Moreover, Finley et al. described the use of microcoils for the localization of pulmonary nodules, which was, however, burdened by a considerable risk of dislodgment [19]. 

The need for the availability of a hybrid operating theater for surgery guided by fluoroscopy represents the major disadvantage of this technique [41]. 

Recently, multiple dyes and contrast agents have been investigated to resolve the drawback of the migration of metallic markers. In the literature, the use of methylene blue, Indigo Carmine, Barium, Lipiodol, and Lopamidol has been reported [13]. Unfortunately, the latter three dyes need fluoroscopic guidance during surgery, even if they are associated with a high reported successful localization rate ranging from 93.3% to 100%. Furthermore, acute inflammatory reaction and edema on the surrounding lung parenchyma have been reported by using Barium localization with the possibility of impairing histological diagnosis [42]. Methylene blue is another dye commonly adopted for the localization of lung nodules, with a reported success rate ranging from 93.3 to 100% for peripherical targets. However, the major reported limitations of its use are the rapid diffusion and poor identification in lungs affected by severe anthracosis and the possibility of impairing the correct histopathological assessment of resected specimens [13,43,44]. 

Alternatively, gamma-emitting radioisotopes can be injected, and the labeled nodules are detected intraoperatively using a gamma probe [34]. 

Alternatively, gamma-emitting radioisotopes can be used with the target lesions, which are intraoperatively detected using a gamma probe [34]. Specific radioactive tracers, such as 99 m Technetium, are feasible for the preoperative marking of pulmonary nodules, but specialized equipment is required, and radiation exposure represents a problem for all radioisotopes [45]. Each of these techniques is currently used in clinical practice with unique disadvantages.

Chul Hwan Park et al. published a systematic review and meta-analysis including 46 studies comparing the efficacy and safety of three pulmonary nodule localization methods for VATS resections: hook-wire, microcoil localization, and lipiodol localization. They concluded that all three localization methods yielded similar highly successful targeting rates; however, the hook-wire localization had a relatively lower success target rate because of dislodgement or migration [13]. 

Kleedehn et al. reported a comparative study between hook-wire needle localization and methylene blue dye marking, and they concluded an equivalent preoperative localization rate with a higher complication percentage of pneumothorax and parenchymal bleeding when using a hook-wire needle. The consequent dislodgement of the wire may cause greater difficulty in finding the lesion, thus requiring thoracotomy [16]. 

Anayama et al. first demonstrated, in an animal study, that he near-infrared fluorescence of ICG injected into the lung at a depth of 20 mm can be detected by near-infrared thoracoscopy [46]; then they compared CT-guided percutaneous marking and bronchoscopic marking using ICG to localize small pulmonary nodules, and they obtained a good success rate in both techniques with a focus on bronchoscopic injection, which can be especially useful for marking multiple small-size nodules [47]. 

In the same way, Yang et al. compared the feasibility and detectability of CT-guided percutaneous marking and the electromagnetic navigation bronchoscopic marking of small-size pulmonary nodules [28].

Zhang et al. in 2019 published the first Chinese-based series of patients with subpleural small nodules localized with CT-guided ICG injection; during VATS resection, 3 out of 35 patients (11.4%) failed to localize the nodules with two unclear fluorescences and one undetected nodule [30].

Recently, Ding et al. published the largest series of patients who underwent VATS surgery for small lung nodules previously detected with ICG injection under CT guidance. They compared the efficacy and safety of CT-guided hook-wire and ICG localization using a mixture of 25 mg indocyanine green with 50 mL of Iopamiro. Through a propensity-score-based balancing analysis, they confirmed that the ICG strategy is superior to the localization procedure based on the preoperative positioning of a hook wire, considering a lower complication rate, a lower reported pain score, and a relatively higher success rate [33].

Gkikas et al. in 2022 published a systematic review and meta-analysis focusing on ICG localization procedures for the detection of lung neoplasms; they considered 30 eligible studies that included patients who underwent the ICG localization of lung nodules by indifferently adopting CT-guided, endobronchial, and intravenous techniques. They confirmed that the CT-guided procedure was associated with overall and median success rates, respectively, of 97.3% and 94.3% and an estimated accuracy at 97.3%. The reported false negative rate was 2.4%, and it was a consequence of a localization failure that is commonly due to ICG spillage and subsequently diffused dye in the chest cavity [48]. 

Currently, there is no gold standard procedure for preoperative localization in clinical practice. 

Our technique presents several specific advantages common to the others illustrated above [49]. The first is the reproducibility and the absence of a learning curve for an interventional radiologist who is already familiar with CT-guided procedures within the lung (e.g., biopsy). Furthermore, ICG injection is well tolerated by the patient, who can freely move free of pain and without the risk of any landmark dislocation. 

Compared to ICG, other dyes, such as methylene blue and lipiodol, have the disadvantage of diffusing rapidly from the site of injection, hampering a precise resection. From the recent results of large animal studies in the past and from human phase I clinical trial, the use of ICG coupled with albumin has been shown to be safe and feasible [50,51].

Recent publications using ICG and NIR imaging for lymph node mapping in breast cancer have reported ICG coupled with albumin rather than fresh frozen plasma; ICG can quickly and reliably migrate to draining lymph node basins, permitting rapid and accurate sentinel lymph node identification in a real-time intraoperative setting. They demonstrated that the adsorption of ICG to human serum albumin results in an increased hydrodynamic diameter and has the effect of increasing fluorescence, leading to improved lymph node detection [50]. For these reasons and considering the potential risks of blood transfusion reactions and infection with fresh frozen plasma, we have chosen albumin as a good coupling agent; our findings have confirmed the experience of Gilmore et al., who first demonstrated that ICG diluted in human albumin increased the fluorescence in the lung parenchyma as well [51]. More studies are necessary to assess the optimal dosing of the ICG mixture to detect lung nodules with a maximum diameter < 25 mm; starting from the previous experiences to detect sentinel lymph nodes in NSLC, we performed a dose-escalation study, and the best results were obtained with the injection of 0.1 to 0.5 mL of 2.5 mg/mL mixture of ICG/albumin depending by the dimension and the depth of the nodule. 

Finally, ICG does not require immediate surgery after the injection because the marking persists from hours to days [46], allowing for more flexibility in the surgical schedule. 

On the other hand, ICG staining may represent some problems and presents some disadvantages. In cases of severe pulmonary emphysema, there is concern that liquid markers may not form a distinct spot but rather diffuse inside pulmonary cysts; therefore, ICG may not be ideal in such cases. 

In addition, ICG staining may represent some drawbacks: in cases of severe pulmonary emphysema, liquid markers may diffuse inside pulmonary bullae, causing a spread of the tracer. 

Moreover, the VATS equipment required to visualize ICG is expensive and not widely available. 

Moreover, the thoracoscopic camera required to identify ICG is more expensive than the standard camera and not always available in all institutions.

## 5. Conclusions

CT-guided marking using ICG-diluted human albumin was shown to be safe and effective for the preoperative localization of small lung nodules.

This technique, which requires skilled interventional radiologists, may overcome the limitations of the currently most adopted marking approaches and has the potential to facilitate accurate and minimally invasive resection of challenging nodules during VATS surgery. However, our population is too limited to define it as the new standard procedure for the localization of small lung nodules during VATS resection, and more studies are necessary to assess the optimal dye marking and its dosing. 

## Figures and Tables

**Table 1:** Patients’ characteristics.

Characteristics	Value
Total patients (*n*)	40
Nodules (*n*)	43
Gender	
Male	23 (57.5%)
Female	17 (42.5%)
Age (years), mean (range)	62.6 (35–83)
Smoking status	
Active	10%
Former	35%
No	55%
FEV1 (liter), mean (range)	2.78 L (1.38–4.92)
FEV1 % (mean), (range)	93.25% (46–125)
Lesion diameter (mm), mean (range)	10.4 mm (3–21)
Distance to pleural surface (mm), mean (range)	8.1 mm (2–28)
Nodule feature	
Solid nodule	36 (83.7%)
Sub-solid nodule	3 (7%)
Pure ground-glass nodule	4 (9.3%)
Surgical margins of resection (mm), mean (range)	13.1 mm (2–23)
Resection time (min), mean (range)	52.17 min (22–119)
Timing to surgery (min), mean (range)	365 min (120–990)
Location of the nodules	
Right upper lobe	9 (20.9%)
Right middle lobe	4 (9.4%)
Right lower lobe	6 (13.9%)
Left upper lobe	9 (20.9%)
Left lower lobe	15 (34.9%)
Final diagnosis	
Metastatic cancer	23 (53.5%)
Non-small lung cancer	10 (23.2%)
Benign lesions	10 (23.2%)

FEV1: Forced expiratory volume in the 1st second.

**Table 2:** Detailed information of lesion and surgery.

N. Case	Gender	Age	Tumor Localization	Tumor Size (cm)	Pathological Closest Resection Margin (cm)	Lung Resection	Pathological Diagnosis
1	M	64	LUL, LLL	1(U); 1.5(L)	1(U); 0.1(L)	Wedge	Metastasis from colorectal tumor
2	F	77	RLL	2.1	0.9	Wedge	Metastasis from colorectal tumor
3	M	61	RUL	1	4.5	Lobectomy	Lung solid adenocarcinoma
4	F	65	RUL	0.7	0.4	Wedge	Lung hamartoma
5	M	60	RUL	0.8	2.3	Wedge	Metastasis from colorectal tumor
6	M	68	LLL	1.2	2	Wedge	Metastasis from colorectal tumor
7	M	64	LLL	1.2	-	Lobectomy	Lung solid Adenocarcinoma
8	M	59	RUL	1.5	1.4	Wedge	Minimally invasive adenocarcinoma
9	M	75	RUL, LLL	0.9(R); 0.8(L)	0.7(R); 0.9(L)	Wedge	Metastasis from kidney tumor
10	M	61	RLL	1	1.2	Wedge	Lung hamartoma
11	F	52	RUL	0.9	0.8	Wedge	Metastasis from colorectal tumor
12	M	35	RLL	1.7	1.3	Wedge	Metastasis from colorectal tumor
13	M	66	LUL	1	3	Wedge	Pulmonary sarcoidosis
14	F	72	LUL	1.2	-	Lobectomy	Lung solid adenocarcinoma
15	F	59	LLL	1	1	Wedge	Metastasis from colorectal tumor
16	M	49	LLL	1.1	2	Wedge	Metastasis from extragonadal germ cell tumor
17	M	79	RML	1.5	4	Wedge	Lung squamous cell carcinoma
18	M	83	RML	0.6	0.3	Wedge	Metastasis from cholangiocarcinoma
19	F	62	LUL	1.7	1.8	Wedge	Metastasis from adenoid cystic carcinoma
20	M	76	RLL	0.7	2	Wedge	Minimally invasive adenocarcinoma
21	F	76	LLL	2.1	1.5	Wedge	Minimally invasive adenocarcinoma
22	F	43	LUL	0.6	0.5	Wedge	Metastasis from melanoma
23	M	74	RUL	1.6	0.4	Wedge	Minimally Invasive Adenocarcinoma
24	M	69	LUL	0.7	1	Wedge	Metastasis from colorectal tumor
25	F	71	LLL	0.5	4	Wedge	Inflammatory nodule
26	F	42	RML	1.3	0.3	Wedge	Metastasis from colorectal tumor
27	M	78	LUL	1.5 0.8	0.5 0.6	Wedge	Bone metaplasia
28	F	52	LLL	0.5	1	Wedge	Neuroendocrine cell’s hyperplasia
29	M	72	RLL	1.2	0.3	Wedge	Metastasis from melanoma
30	F	57	RLL	0.6	0.8	Wedge	Inflammatory nodule
31	F	61	LLL	0.7	0.6	Wedge	Metastasis from lung squamous cell carcinoma
32	M	46	LLL	0.3	1.2	Wedge	Metastasis from adrenal gland tumor
33	F	41	RUL	0.9	2.5	Wedge	Metastasis from colorectal tumor
34	M	52	RML	0.8	1.5	Wedge	Metastasis from colorectal tumor
35	F	52	LLL	0.6	0.7	Wedge	Inflammatory nodule
36	M	83	RUL	0.6	0.9	Wedge	Minimally invasive adenocarcinoma
37	M	76	LLL	1.9	1.2	Wedge	Metastasis from colorectal tumor
38	F	73	LLL	0.8	1.1	Segmentectomy	Metastasis from colorectal tumor
39	M	50	LLL	1.1	0.5	Wedge	Metastasis from colorectal tumor
40	F	49	LUL	0.9	1.5	Wedge	Lung hamartoma

RUL, right upper lobe; RML, right medium lobe; RLLL, right inferior lobe; LUL, left upper lobe; LLL, left lower lobe; R, right; L, left.

## Data Availability

Derived data supporting the findings of this study are available from the corresponding author (E.V.) on request.
